# Photothermal conversion of biodegradable fluids and carbon black nanofluids

**DOI:** 10.1038/s41598-022-07469-w

**Published:** 2022-03-01

**Authors:** Anna Kosinska, Boris V. Balakin, Pawel Kosinski

**Affiliations:** 1 Department of Mechanical and Marine Engineering, Western Norway University of Applied Sciences, Bergen, Norway; 2 Department of Thermal Physics, National Research Nuclear University MEPhI (Moscow Engineering Physics Institute), Moscow, Russia; 3 Department of Physics and Technology, University of Bergen, Bergen, Norway

**Keywords:** Energy science and technology, Renewable energy, Solar energy, Solar thermal energy

## Abstract

The paper is devoted to the topic of direct absorption solar collectors (DASCs). Various kinds of fluids can be used as heat transfer fluid in DASCs, and the main focus of our paper is on comparing nanofluids (water with carbon black nanoparticles, concentrations between 0.25 and 1.00% weight) and biodegradable coffee colloids. At first, these fluids were tested by exposing them to irradiation caused by artificial light in indoor experiments, and the corresponding temperature increase was recorded. The fluids were placed in a beaker with a relatively large size so that most of the fluid was not directly irradiated. In these experiments, the performance of the two studied fluids was similar: the resulting temperature increase varied between 46 and 50 °C. Our next experiments involved a smaller system subjected to irradiation obtained by using a solar collector. As a result, we detected an intense absorption on the nanoparticle surface so that the temperature rise in the nanofluid was higher than in the coffee colloids. Next, the process was analysed using a theoretical analysis that gave good correspondence with the experiments. Finally, we extended the theoretical analysis to a DASC with a flowing fluid. The model was validated against results from the literature, but it also supported our experimental findings.

## Introduction

Different techniques within solar thermal technology can be used to convert energy from the sun into heat. One of them involves direct absorption solar collectors (DASCs), which utilise fluids subjected to solar irradiation (see e.g. Gorji and Ranjbar^1^). The heated fluids are later transferred further into the system. As such, the solar thermal technology differs from the photovoltaic techniques that produce electricity.

Water is perhaps the most obvious choice as a heat transfer fluid in DASCs. There have, however, in the research literature been frequent attempts to replace water with fluids possessing superior properties. Here the appropriate candidates are “black fluids”, that is, liquids that offer better absorption of solar radiation. The paper by Minardi and Chuang^2^ was, perhaps, the first that investigated this issue. In their work, they considered ink in water. More recent research papers, however, usually exploit fluids with suspended nanoparticles (so-called nanofluids).

Different types of nanofluids were investigated in the literature. Some examples are the following systems: copper-water^3,4^, aluminium oxide-water^5–7^, $\text {TiO}_2$, $\text {SiO}_2$, CuO, or carbon nanotubes in water and in oil^8^. In our previous research, we studied carbon black (CB) nanoparticles^9–11^ and iron oxide nanoparticles^12,13^ in water.

There is, however, another alternative, namely the use of biodegradable fluids. An interesting example is coffee colloids, which are widely available, inexpensive and eco-friendly. Moreover, they function without other drawbacks inherent to metallic nanofluids such as potential erosion^14–16^ or nanoparticle deposition/clogging. Nevertheless, this topic has only been discussed in a few papers^11,17,18^. Therefore, they are the objective of this research.

In this paper, we aim to compare carbon black nanofluids with coffee colloids regarding potential applications in solar thermal energy. Our research was structured as follows. First, a stationary system consisting of a beaker filled with a nanofluid/coffee colloid was studied. The beaker was subjected to irradiation by a halogen lamp that mimicked the solar radiation. This was done in a laboratory under controlled conditions. Different concentrations of the nanofluids were considered, and they were compared with the coffee colloids. This part of the paper resembles our previous paper^11^, but this time we show that the change of the set-up geometry has a significant influence on the conclusions.

In the second step, a similar set-up was subjected to real solar irradiation in field experiments. In addition, a parabolic solar collector was used to concentrate the light. This resulted in a high temperature increase; moreover, the conclusions were not the same as for the previous experiments with a halogen lamp.

In the third step, we conducted a theoretical analysis of the studied process. At first, a simplistic model was suggested and validated against our laboratory experiments. Finally, a similar model was applied for studying a simple model of a DASC and validated against results from the literature. It was shown that the choice of heat transfer fluid does not need to play an important role and the biodegradable coffee colloids may replace the CB nanofluids in some applications. It is also interesting to note that our simplistic mathematical model leads to results that are comparable with experiments.

## Analysis of stationary fluids: indoor and field experiments

### Fluid preparation

**Figure 1:**
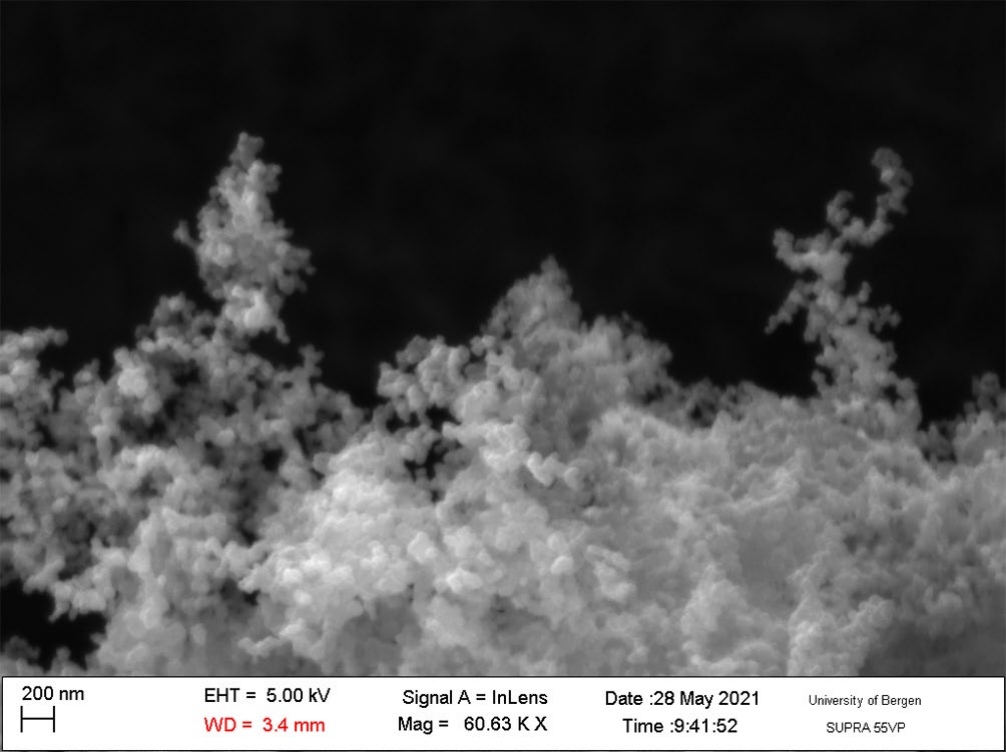
Scanning electron microscopy image of the carbon black nanoparticles investigated in this research.

There are two kinds of fluids studied in this research. The first type was a nanofluid produced by dispersing CB in distilled water. CB was of type Timcal Ensaco 350G, and an image of the CB nanoparticles is shown in Fig. 1. This was obtained using a scanning electron microscope of the type Zeiss SUPRA 55VP S.

A weighted sample of CB was mixed with distilled water, together with the same amount of sodium dodecyl sulfate that was necessary to stabilise the nanofluid. At first, the suspension was mixed using a standard ceramic magnetic stirrer. The duration of the process was 20 min. Afterwards, a beaker with the suspension was placed in an ultrasonic bath of the type Branson 3510. The duration of the sonication was 1 h. It must be noted that a similar procedure was used in our previous works^9–11,13^, as well as by other researchers^19–22^.

We produced three different weight concentrations of CB nanofluid: 0.25%, 0.50%, 1.0%. Thus, this research differed from our previous paper^11^, where the focus was on higher concentrations.

**Figure 2:**
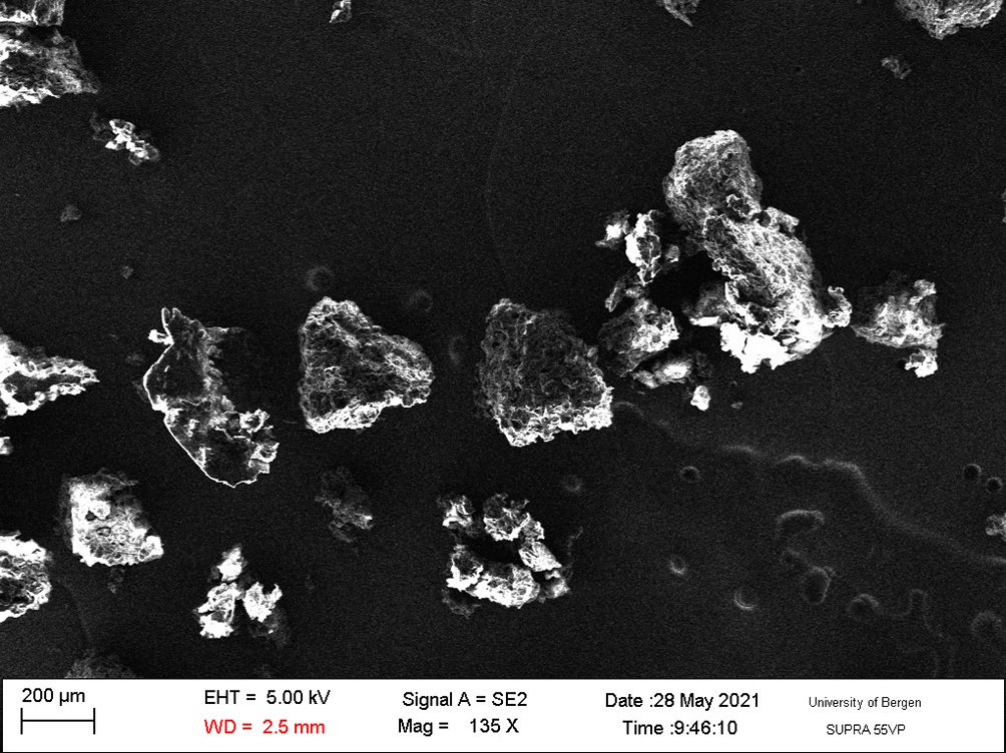
Scanning electron microscopy image of the dry coffee particles.

**Figure 3:**
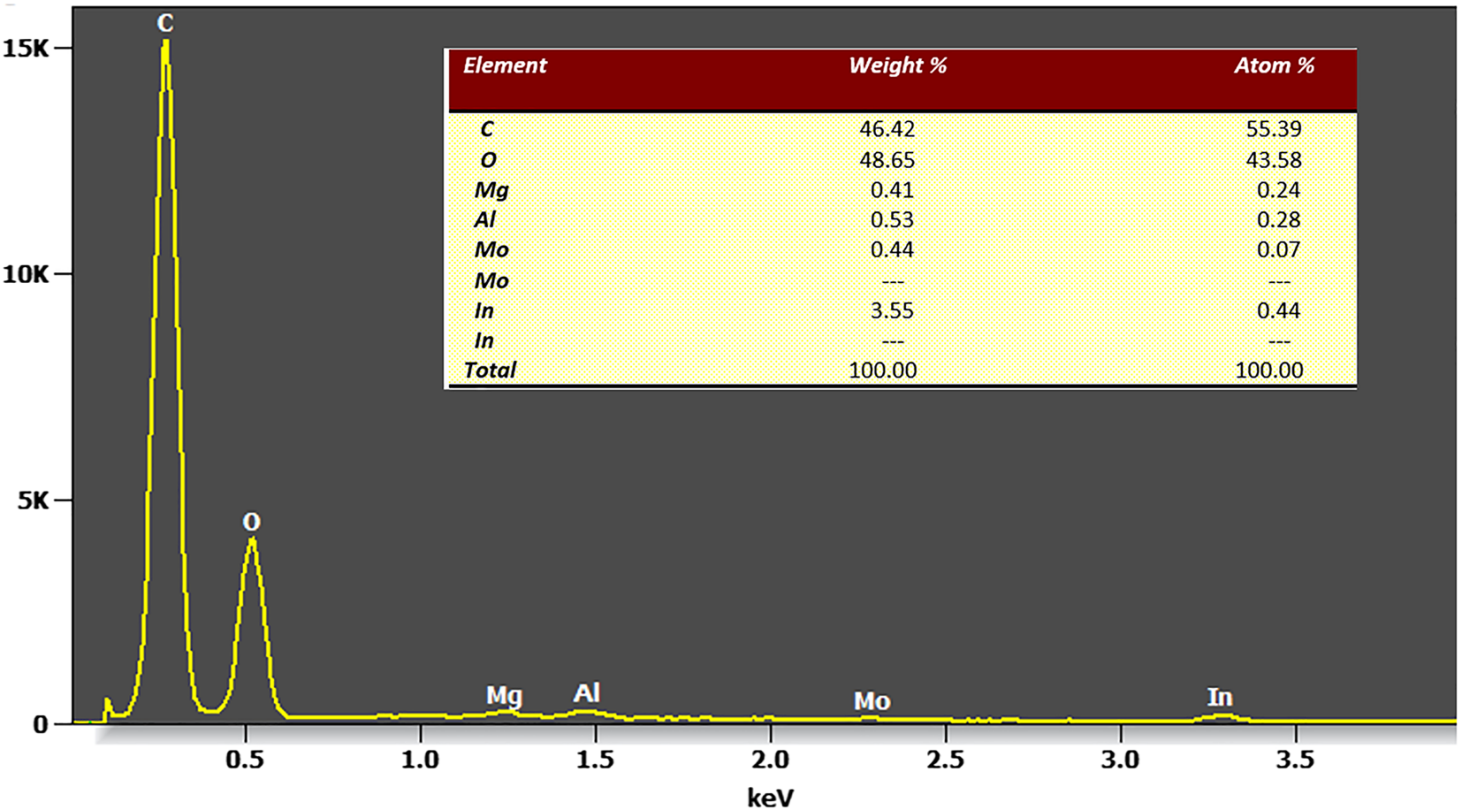
Energy-dispersive X-ray spectroscopy of the coffee particles. The most dominant elements were carbon and oxygen.

The second fluid was a coffee colloid obtained from Arabica coffee of the Tchibo brand. Figure 2 shows a SEM image of the dry coffee powder, and Fig. 3 shows a spectrum acquired via energy-dispersive X-ray spectroscopy (EDX).

Similar to our previous research^11^, the coffee colloids were produced using a standard coffee brewer. The brewing process was run three times to maximise the extraction process. In our previous paper, different concentrations were tested and the main conclusion was that a simple increase in concentration leads to better results regarding the irradiation absorption. Therefore, in the present paper, we selected the highest concentration tested in^11^, which was 15.0 g coffee powder dispersed in 300 ml of water.

### Indoor experiments

The objective of the first part of the experiments was to investigate a relatively simple case, where a studied fluid (coffee colloid or nanofluid) was subjected to halogen lamp irradiation in a laboratory. The halogen lamp mimicked the solar irradiation. According to our previous research^23^, the spectrum of the lamp differs from the solar spectrum by increasing the wavelength corresponding to the maximum radiating power by around 350 nm. Despite this difference, the obtained results can later be tuned by using more advanced experimental set-ups and real solar irradiation. Therefore, in the next section of the paper, we also show results obtained during outdoor experiments.

**Figure 4:**
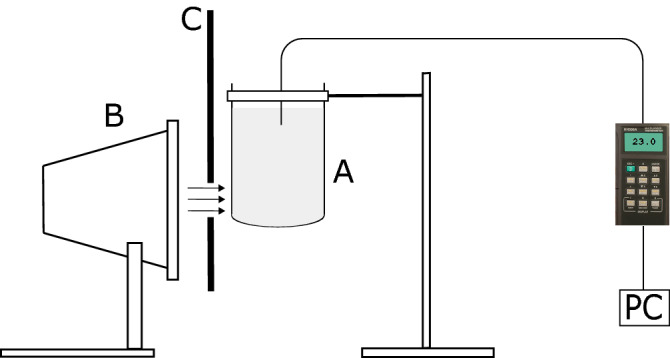
The schematic of indoor experiments (not to scale). A beaker with a studied liquid is subjected to halogen light (the figure was created using Inkscape version 0.92).

Figure 4 shows a schematic of the experimental set-up. The fluid was placed in a beaker (denoted as A) whose inner diameter was 56 mm; the wall thickness was 2 mm, and the height was 120 mm. The height of the fluid was 105 mm. The beaker with the fluid was subjected to irradiation from the halogen lamp (B) that was located behind a protective and insulating screen (C). The height of the slot in the screen was 3 cm, and the distance between the light source and the beaker was 4 cm.

The temperature was measured by a thermocouple via a multi-logger thermometer of type Omega HH506RA. The thermocouple was immersed at the depth of 20 mm. During preliminary experiments, we tested different locations of the thermocouple, but the influence on the results was minor (the difference did not exceed 0.5 K).

This part of the research resembles our previous investigated reported in^11^, as well as in^9,10,24^. There are, however, some important differences. At first, we used a beaker of different geometry. Also, the distance between the light source and the beaker was less than in^11^. The main objective was to check whether this change of design parameters will result in a similar performance.

**Figure 5:**
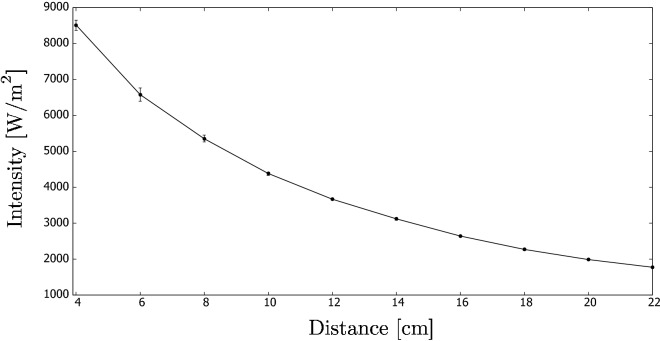
Intensity of the halogen lamp vs. distance.

The halogen lamp was a Cotech 400WV floodlight with an Osram light bulb. Figure 5 shows the intensity of the light irradiation measured as a function of distance from the lamp. The measurement was performed using a sensor from Linshang Technology. The error bars in the figure depict the spread of the measurements, where we collected our experimental results. An average was denoted by the dot. We used the same strategy in Figures 8 and 14 discussed later in the paper.

**Figure 6:**
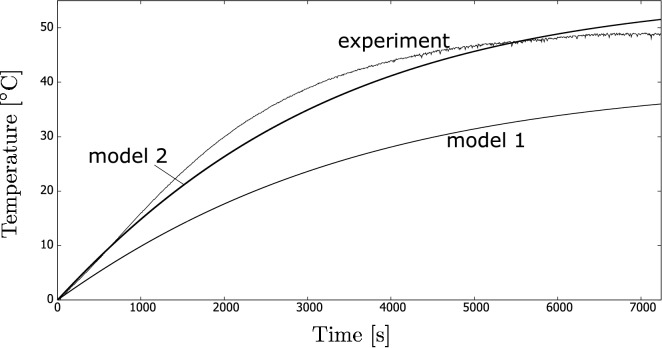
History of temperature increase (an example) for the coffee colloid sample during the indoor experiments. The results also validate the two mathematical models discussed in “Theoretical analysis” section.

**Figure 7:**
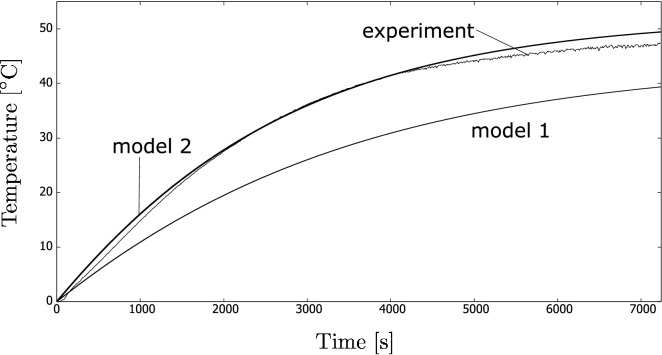
History of temperature increase (an example) for a nanofluid sample during the indoor experiments. The results are compared with the theoretical analysis discussed in “Theoretical analysis” section.

The fluid samples were irradiated for 7200 s. An example of temperature increase history is shown in Fig. 6 (denoted as “experiment” in the graph). Similarly, Fig. 7 shows an example of temperature increase history for one of the nanofluid samples. It must be noted that the figures show histories of only one measurement, that is, an unsteady process. Therefore, we do not show the mean results yet.

**Figure 8:**
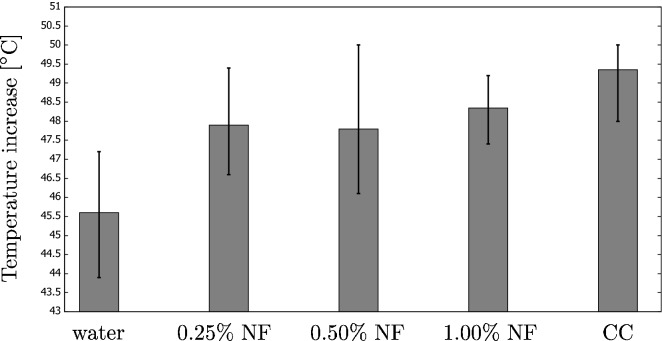
The temperature increase for different fluids involved in the indoor experiments (NF: nanofluid; CC: coffee colloid). All the fluids (except for distilled water) result in a similar performance.

We repeated each experiment at least four times, and Fig. 8 shows the final increase in temperature. This was recorded after 7200 s (when the process became steady). The figure compares different concentrations of the CB nanofluid with the coffee colloid.

The main observation is that the temperature increase is similar for all the concentrations of the nanofluid and coffee colloid. This differs from our previous paper, where the nanofluids outperformed the coffee colloid. For instance, the thermal efficiency was about 2% greater for carbon black nanofluids if compared to the coffee colloids with the highest concentration. There are, however, important differences in the design of the experimental setup. In the present research, the irradiated area (if compared to the fluid height) was larger than the one used in our previous paper. Also, the intensity of radiation was higher due to the lower distance between the halogen lamp and the beaker. These two factors may have resulted in a greater heat loss to the surroundings.

In addition, the diameter of the beaker was larger if compared with our previous research so that most of the fluid may have stayed in a region not directly irradiated. Thus, the final temperature increase is also less dependent on the fluid properties.

This also shows that choice of a type of fluid and its concentration may be outperformed by the design of a DASC. In other words, it may be challenging to find an optimal fluid without considering its practical use. Later in the paper, we discuss this issue more when theoretically investigating at performance of a simple DASC.

### Field experiments

**Figure 9:**
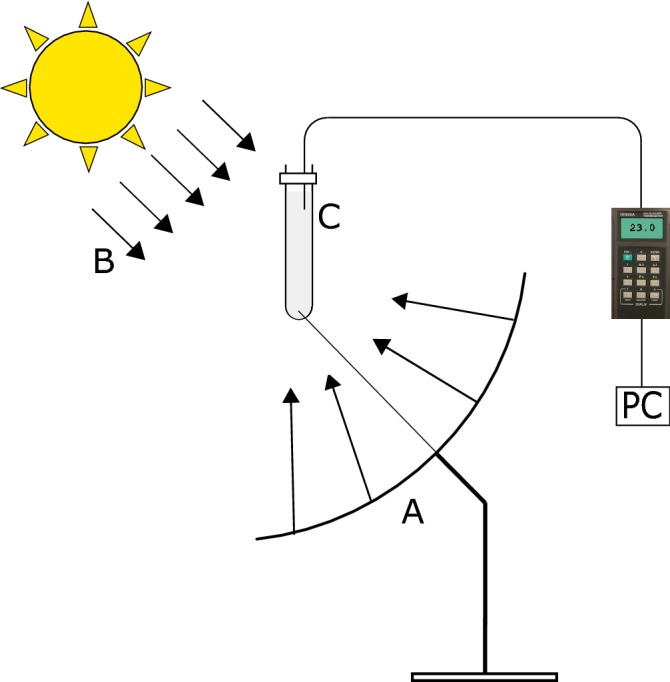
The schematic of the field experimental rig (not to scale). A beaker with a studied liquid is subjected to concentrated solar irradiation. The beaker is located at the focal point of a parabolic collector (the figure was created using Inkscape version 0.92).

The second series of experiments was performed under real solar conditions. The nanofluid and coffee colloid were placed in two separate glass beakers and subjected to concentrated solar irradiation by using parabolic solar collectors. The schematic from one of the two set-ups is shown in Fig. 9, where the solar collector is denoted as A, the solar radiation as B and the beaker as C. We used two identical parabolic solar collectors with a diameter of 30 cm, with the studied fluids located in the focal points.

The objective was to run these two tests simultaneously under the same weather conditions. It must, however, be noted that the solar collectors may still have differed, especially in the light reflectivity from their surfaces. Furthermore, the results were significantly influenced by a changing wind speed during the measurements.

The beakers had an inner diameter of 21 mm and a thickness of 1.5 mm. The length was 150 mm with the fluid height equal to 120 mm. Thus, we used smaller beakers than in the indoor experiments (larger beakers could significantly shield the solar collectors from solar radiation). The temperature was measured in the same way as during the indoor experiments.

We selected only one concentration of CB nanofluid, namely 0.5% because the indoor tests did not reveal any important differences between the concentrations, and since our main objective was rather to compare the performance of nanofluids vs. coffee colloids.

The experiments were performed close to the city of Bergen (western Norway) at $60^{\circ }$18′41.2″N, $5^{\circ }$20′42.0″E in May and June in 2021, around noon. Only sunny days were selected for the experiments.

We did not use any automatic solar tracking devices to adjust the orientation of the collectors. Nevertheless, the duration of each test was short (around 10–15 min). During this period the angular motion of the sun did not exceed 4°. This allowed us to manually set the focal point on the beaker. Also, we ran four tests on different days to assure repeatability. As a result all the tests led to the same conclusions as shown in the following. This indicated that this simple technique could be applied in our measurements.

**Figure 10:**
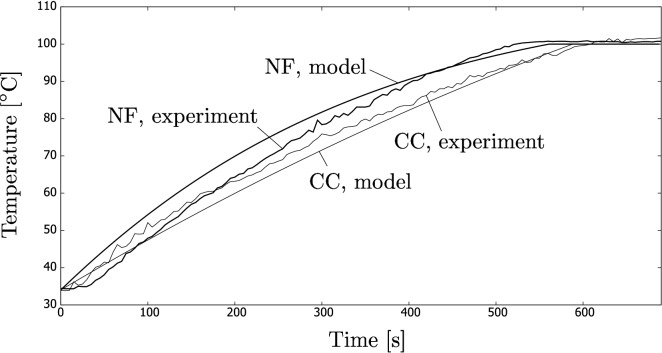
A typical history of fluids temperature during the field experiments using the solar concentrators. The results are compared with the theoretical analysis discussed in “Theoretical analysis” section.

Figure 10 shows a history of temperature increase in the nanofluid and coffee colloid. An interesting observation is that the process is much more rapid than it was for the indoor experiments because the beam of the concentrated light was much more intense. In addition, the amount of fluid was also less in the field experiments. As a result, the boiling temperature was reached after a few minutes.

In the case of nanofluids, we detected a crackling sound during the process. Most probably, this was caused by implosions due to a subcooled water boiling at the CB particles, something not observed for the coffee colloids. This indicates that the process of radiation absorption in nanofluids is more complex and occurs locally on nanoparticle surfaces. The phenomenon did not happen during the indoor experiments, where the light was not concentrated so that its intensity was significantly lower.

A summary of all the four experiments is shown in Table 1. The first two columns show the weather conditions at the beginning of each test (the solar irradiation and air temperature). This was obtained from the local weather station (Ytrebygda Weather Station). The next two columns depict the initial temperatures of the fluids, i.e., before the beakers were placed in the focal points of the collectors. The temperatures were always higher than the ambient temperature because the samples were allowed to stay under the sun until their temperature stabilised.

The last two columns show the measured time until the boiling commenced, that is, the temperature became $100\;^{\circ }\text {C}$ (also observed visually). It can be clearly seen that the nanofluid reached the boiling temperature faster.

**Table 1:** Outdoor results.

No.	Solar irrad. (W/m^2^)	Temp. surround. (°)	Init. temp. CC (°)	Init. temp. NF (°)	Time until boil. CC (s)	Time until boil. NF (s)
1	784	$18.0$	$32.2$	$36.3$	945	875
2	744	$15.7$	$29.6$	$33.2$	950	665
3	775	$16.4$	$30.7$	$32.4$	760	745
4	768	$25.7$	$33.9$	$34.4$	610	525

An interesting conclusion that can be drawn is that nanofluids are better candidates for the solar thermal energy technology if concentrated solar beams are used. This is due to the subcooled boiling.

## Theoretical analysis

The objective of this section is to present a mathematical model and computational results that can later be used to speculate on the potential use of biodegradable fluids in DASCs. At first, however, we pay attention to the results of the rig irradiation and show a tool that can be potentially used for the modelling of this system. In the next sections, our model is further extended to the modelling of DASCs.

The performance of the indoor rig can be easily described using the theoretical analysis presented in our previous paper^11^. In the following, we recapitulate the main steps of the model. In addition, we modify the model so that it better corresponds to the experimental observations.

**Figure 11:**
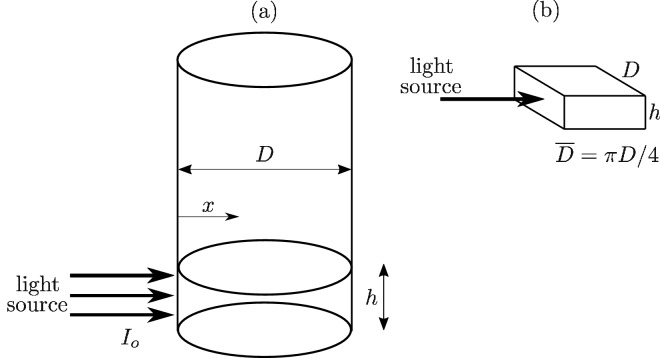
Theoretical analysis of the indoor set-up (the figure was created using Inkscape version 0.92).

Figure 11 shows a schematic of the studied beaker with a zone (height *h*) that is directly irradiated. We assume that *h* is equal to the size of the slot in screen B (from Fig. 4). We also state that the irradiated zone can be modelled as a cuboid (Fig. b), according to^11^. The size ${\overline{D}}$ was derived in^11^.

We begin the analysis with the simple energy balance:

1 $$\begin{aligned} mc\frac{dT}{dt} = q - \alpha A(T-T_o) - A \varepsilon \sigma (T^4-T_o^4), \end{aligned}$$

where *m* is the fluid mass, *c* is its heat capacity, *T* and $T_o$ are the current fluid temperature and temperature of the surroundings, respectively. Furthermore, *q* is the absorbed heat due to the light, $\alpha$ is the heat transfer coefficient, *A* is the total area of the beaker, $\sigma$ is the Stefan-Boltzmann constant, and $\varepsilon$ is emissivity.

Next, we use Beer-Lambert’s law^1,25,26^ to calculate the volumetric heat generation within the fluid:

2 $$\begin{aligned} I(x) = I_o \exp (-Kx) \Rightarrow q_v (x) = -\frac{dI}{dx} = I_o K \exp (-Kx), \end{aligned}$$

where *K* is the extinction coefficient, and $I_o$ is the incident irradiation intensity. The total heat generation in the whole irradiated zone can be found by the integration of the volumetric heat generation $q_v$ between *x* = 0 and *x* = ${\overline{D}}$ (see also^11^):

3 $$\begin{aligned} q = I_o D h \left[ 1-\exp (-K{\overline{D}})\right] . \end{aligned}$$

In the above, $I_o$ can be found by interpolation of Fig. 5 so that it is calculated for the distance that is equal to $b + (D/2 - \pi D/8)$^11^, where *b* = 4 cm in this research (i.e., the distance between the lamp source and the edge of the beaker).

Thus, we obtain a model that is called Model 1 in the following. This model is fully based on our previous work^11^.

Nevertheless, the model can be extended by assuming that the incident intensity is not constant but varies with distance due to the circular shape of the beaker. In the subsequent sections, this extended model is called Model 2.

First, $I_o D h$ from Eq. (3) can be understood as the incident heat on the cuboid surface. This part of the equation can be replaced by (with $R=D/2$):

4 $$\begin{aligned} I_o D h \equiv 2h \int _{0}^{\pi /2}I(y) R \mathrm {d}\phi , \end{aligned}$$

that is, only one half of the circular side is irradiated. The curve in Fig. 5 can be approximated by a second grade polynomial so that:

5 $$\begin{aligned} I_o D h \equiv 2 h R \int _{0}^{\pi /2} (aR^2\sin ^2 \phi + bR \sin \phi +c) \mathrm {d}\phi , \end{aligned}$$

where $a = 217794 \,\text {W}/\text {m}^4$; $b = 61541 \,\text {W}/\text {m}^3$, $c = 6290 \,\text {W}/\text {m}^3$ when using our lamp. Hence, Eq. (3) becomes:

6 $$\begin{aligned} q = 19.2 \left[ 1-\exp (-K{\overline{D}})\right] . \end{aligned}$$

Equation (1) was then solved numerically using an explicit Euler scheme. The initial and ambient temperatures were the same in the model.

The extinction coefficient for the investigated coffee colloid was estimated to be $53 \,\text {m}^{-1}$^11^. This was found using the halogen lamp, thus it may not directly correspond to the solar spectrum. Nevertheless, we assume that it will not change significantly. Also, the extinction coefficient for CB nanofluids is a few orders of magnitude higher, i.e., between $10^4$ and $10^5\, \text {m}^{-1}$^26^. Therefore, we assumed this coefficient to be $5\cdot 10^4\,\text {m}^{-1}$ for our calculations.

The heat transfer coefficient $\alpha$ was estimated according to empirical relations^27^ for the Nusselt number. Using the geometry of the beaker and the surrounding air properties, we found:

7 $$\begin{aligned} \alpha = \beta \cdot 2.72 (T - T_o)^{0.25}, \end{aligned}$$

where $\beta$ is a correction factor. We introduced it to the model to account for differences between empirical relations from the literature and our set-up. The value of emissivity was selected to be equal to 0.8 (carbon). Thus, $\beta$ considers also potential discrepancies between this value and reality.

The results for the coffee colloids, where Models 1 and 2 were used, are shown in Fig. 6. The figure shows a history of the temperature increase from the initial one for the case where the coffee colloid was used. The results are compared with one of the experiments. For both models, $\beta$ was selected to be 1.0, i.e., no correction was done. An analogous result is shown in Fig. 7 for the case of the nanofluid. The correction parameter $\beta$ was 1.4 for Model 2. Similarly to the above case, $\beta$ was equal to 1.0 for Model 1.

The new model (Model 2) better matches the experiments. The rate of the temperature increase is more underestimated in Model 1. During the initial stage, the heat loss plays a minor role so that the choice of the correction coefficient $\beta$ was less important. We must emphasise that these correction parameters may be case dependent (i.e., its choice may be treated as questionable), and it is thus interesting that there is still a rather satisfactory correspondence between the theory and experiments.

A similar mathematical model can also be developed for the outdoor experiments. In this research, we assumed that the beaker with a studied fluid is subjected to concentrated light irradiation with intensity:

8 $$\begin{aligned} I_o = \gamma \frac{A_c}{A_s} I_{sun}, \end{aligned}$$

where $A_c$ is the area of the parabolic solar collector, $A_s$ is the area of the irradiated zone, and $I_{sun}$ is the sun irradiation. The parameter $\gamma$ accounts for the inefficiency due to the limited reflectivity of the collector, heat losses, shading of the collector by the rig, reflection from the glass beaker, inaccuracies when placing the beaker in the focal point, the limited accuracy of estimating the size of the irradiated zone and so on. We selected the parameter $\gamma$ to be 0.8 because it validated our experimental results well

Equation (3) can be re-written to:

9 $$\begin{aligned} q = I_o A_s \left[ 1-\exp (-K{\overline{D}})\right] =\gamma A_c I_{sun} \left[ 1-\exp (-K{\overline{D}})\right] . \end{aligned}$$

This shows that the absorbed heat does not depend on the size of the irradiated zone as long as it is smaller than the size of the beaker.

In addition, the heat loss from the beaker to the surroundings was modelled using Eq. (7), and the temperature of the fluid as a function of time was found using Eq. (1). The results of the fluid temperatures are shown in Fig. 10 and compared to the previously discussed results.

For the coffee colloid, the parameter $\beta$ in the heat loss model was equal to 1.0, i.e., as in the literature. Thus, our whole mathematical model describes the real process surprisingly well. For the nanofluid, the parameter had to be increased to 5.5. A reason may be a local high temperature (probably around at least the boiling temperature) that results in an intensive heat loss.

It must also be noted that the mathematical model has one crucial advantage, which is its simplicity. This may indicate that similar models can be potentially exploited for other solar thermal energy applications. This is the topic of the next section.

## Potential use for DASCs

In the present section, we show a theoretical analysis of the use of the coffee colloids and nanofluids in a DASC. The results were compared with results by Bardsgard et al^26^, which were inspired by Otanicar et al^28^.

**Figure 12:**
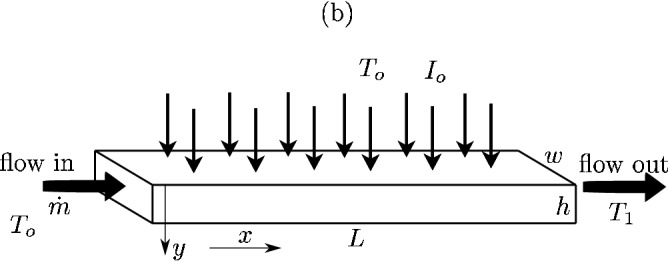
Theoretical analysis of a DASC (the figure was created using Inkscape version 0.92).

We investigated a simple system that mimics a part of a larger DASC. The schematic is shown in Fig. 12. A system with a flowing fluid has length *L*, width *w* and height *h*, and its mass flow rate was ${\dot{m}}$.

In our first analysis, we use the same parameters as investigated in^26^, i.e., *L* = 0.05 m, *w* = 0.03 m, and *h* varied in the simulations. Next, $I_o$ = 1000 W/$\text {m}^2$ (= 1 sun), the velocity was 0.0026 m/s, and the properties of the fluid were the same as for water. The initial and ambient temperatures were $T_o$ = 25 $^{\circ }$C. The heat loss coefficient varied in^26^ between 23 and 34 W/($\text {m}^{\circ }$C). In our research, we selected the highest of these values because it fitted well when comparing the results. The main objective was to find the outlet temperature $T_1$.

The system investigated in^26^ was based on full computational fluid dynamics (CFD) simulations. Thus, the model considered the fluid flow in a domain subjected to solar irradiation and solved numerically. Therefore, our analytic model is significantly simpler.

In our mathematical model, the energy balance was (a steady flow):

10 $$\begin{aligned} {\dot{m}} c (T_1 - T_o) = wLI_o \left[ 1-\exp (-Kh)\right] - \alpha wL(T_{ave}-T_o) - \varepsilon \sigma wL (T_{ave}^4-T_o^4) , \end{aligned}$$

where we assume an average temperature, $T_{ave} = 0.5(T_o + T_1)$, for the heat loss calculation. Nevertheless, the thermal radiation was not modelled in^26^ because it was accounted for by the heat loss coefficient. In such a case, it is then straightforward to find the outlet temperature from Eq. (10).

**Figure 13:**
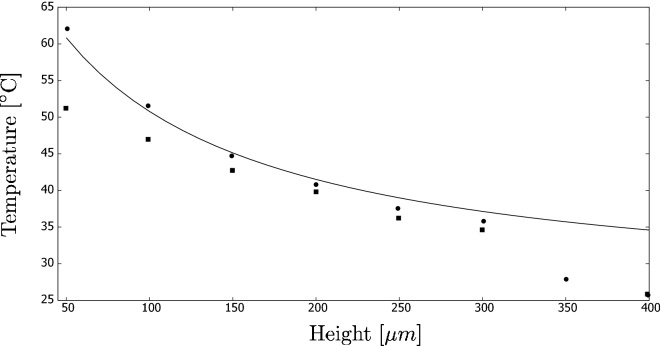
Theoretical analysis of a DASC (solid line) compared with^26^ (boxes and circles). The literature results focus on two different scenarios that were not modelled by us.

Figure 13 shows the results (the solid curve) of the outlet temperature computed for various values of the stream height. The results refer to a CB nanofluid with $K = 5\cdot 10^4\, \text {m}^{-1}$ and are compared to^26^ (the black boxes). It is interesting to note that our simplistic model leads to very satisfactory results, without a necessity to run complex CFD simulations or costly experiments.

Also, the same mathematical model was used for estimation of efficiency of the experimental setup developed by Arberghini et al^17^. Similarly to our paper, these researchers used coffee colloids in a DASC. Despite the complexity of their rig, it could also be simulated using a simplistic system shown in Fig. 12. The geometric parameters were: $L = 0.404 \,\text {m}$, $w = 0.0225 \,\text {m}$, $h = 0.006 \,\text {m}$. Alberghini et al assumed that the wind speed during measurements was 1.0 m/s. Also, they used three different values of the volumetric flow rate of the heat transfer fluid: 0.138, 0.276 and 0.414 ml/s.

In our simulations, we considered the convective heat loss from the upper surface. Using the provided wind speed and empirical relations for the Nusselt number from^27^, we estimated the heat transfer coefficient to be 7 W/($\text {m}^{\circ }$C). Furthermore, we used the value of the extinction coefficient corresponding to our measurements, i.e., $53 \,\text {m}^{-1}$. The efficiency was calculated in the same way as in our previous paper^11^.

Also, the thermal radiation was modelled when simulating the results from Alberghini et al. Since the temperature increase in the studied DASC was low, the third term on the right-hand side of Eq. (10) was linearized. For this, we define $\Delta T = T_{ave} - T_o$ so that $T_{ave}^4 = (T_o + \Delta T)^4 = T_o^4 + 4 T_o^3 + \cdots$. Hence $(T_{ave}^4 - T_o^4) \approx 4 T_o^3$.

**Figure 14:**
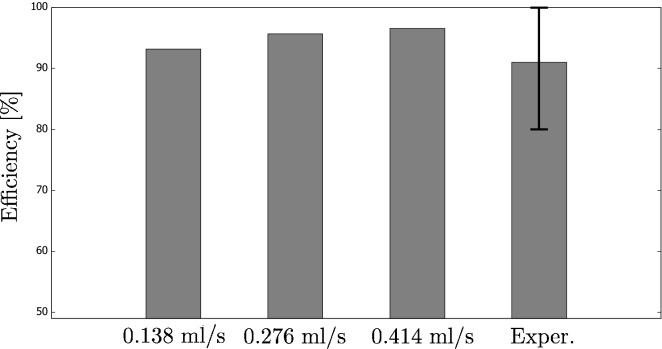
The efficiency calculated using the simplistic model and validated against the experimental results from^17^. The theoretical analysis was performed for three different values of the volumetric flow rate.

The results are depicted in Fig. 14. The three first bars in the histogram show the efficiencies obtained for the three studied values of the volumetric flow rate. The conclusion is that an increase of the flow velocity improves the efficiency as the residence time in the system is lower, so it is less subjected to heat losses. We must emphasize that the same was stated in^17^. The last bar shows experimental results from^17^ that is a mean of the results of the different flow rates and obtained for coffee colloids with the highest concentration. It is interesting to note that our results are similar to^17^. The differences can also be a result of higher thermal losses, e.g., from the other surfaces of the system, as well as other material properties (especially the extinction coefficient).

Finally, we used a similar for a sensitivity analysis of a DASC. For this, the height *h* was significantly increased to 1.0 and 3.0 cm, i.e., to values that correspond better to a real design. Also, the length *L* was similarly increased to 0.5 m. It must, however, be noted that this length increase does not influence the final result, and other values would also be possible. The width *w* was not changed.

**Figure 15:**
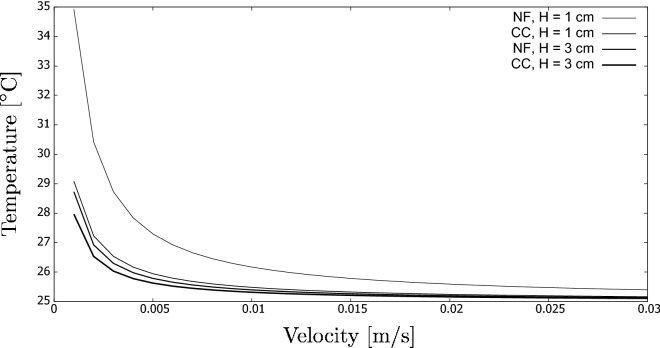
Sensitivity analysis using a simplistic model of a DASC. The graph shows the influence of the system height, kind of fluid (NF: nanofluid; CC: coffee colloid) and flow velocity on the final temperature.

In our computations, we compared the two studied fluids (nanofluids vs. coffee colloids) and the two aforementioned heights. This resulted in four different cases. Figure 15 shows the results in the form of the outlet temperature dependence on the flow velocity.

There are two interesting conclusions that can be drawn here. First, the lowest velocities increase the residence time so that the fluids were subjected longer to irradiation. Nevertheless, this also leads to higher heat losses due to the higher temperature and longer exposure time to the surroundings. Still, in the studied set-up, the heat loss did not manage to decelerate the process significantly.

A more interesting conclusion is that nanofluids are more clearly superior to coffee colloids if the fluid height is low. For the highest value of the height, the results were relatively similar. The reason is that the penetration depth for solar radiation is very low for nanofluids. Thus, most of the absorption occurs in a thin layer of fluid, as also observed by, e.g.,^26^. This may also explain the results observed for our indoor experiments, where the performance of both fluids was almost the same. It must be mentioned, however, that it does not need to be a case for intense radiation that leads to local boiling on the particle surface, as it occurred during our field experiments.

## Concluding remarks

When designing DASCs, attention should be paid to the system geometry so that most of the fluid is directly subjected to solar irradiation. In the present research, this was illustrated by fluid “depth”: for systems where the depth was higher, the final performance did not seem to be dependent on the kind of fluid. On the other hand, the solar irradiation penetration in fluids can be very low, especially for CB nanofluids. Here other kinds of fluids (e.g., biodegradable coffee colloids) should be sufficient because they do not necessarily underperform nanofluids regarding their thermal efficiency.

Nevertheless, this does not need to be the case if concentrated lights are used, for instance, if solar collectors are exploited. The intense beam of light may lead to local subcooled boiling on the particle surface. This leads to a quick local temperature increase, which decreases the heat loss assuming that the heated fluid is promptly evacuated to insulated parts of the system.

It should also be noted that DASCs can easily be theoretically described by using relatively simple mathematical relations, as shown in the paper. Aspects like complex fluid flows, heat transfer and subcooled boiling may be challenging to describe analytically, but still, the results may not be far from reality.
